# Supplementary material for: Antipsychotic-induced extrapyramidal side effects: A systematic review and meta-analysis of observational studies
Source: PLoS One. 2021 Sep 10;16(9):e0257129. doi: 10.1371/journal.pone.0257129 (PMC8432767; doi:10.1371/journal.pone.0257129)
Supplement: S3 Table — The table shows the risk of bias assessments of studies with regard to design, conduct and analysis. (DOCX) [file pone.0257129.s003.docx]

| Author, year | Q1 | | | | Q2 | | | | Q3 | | | | Q4 | | | | Q5 | | | | | Q6 | | | | | Q7 | | | | Q8 | | | | | Q9 | | | | | Q10 | | | | | | Q11 | | | | Overall quality result | |
| --- | --- | --- | --- | --- | --- | --- | --- | --- | --- | --- | --- | --- | --- | --- | --- | --- | --- | --- | --- | --- | --- | --- | --- | --- | --- | --- | --- | --- | --- | --- | --- | --- | --- | --- | --- | --- | --- | --- | --- | --- | --- | --- | --- | --- | --- | --- | --- | --- | --- | --- | --- | --- |
|  | Y | N | U | NA | Y | N | U | NA | Y | N | U | NA | Y | N | U | NA | | Y | N | U | NA | | Y | N | U | NA | Y | N | U | NA | | Y | N | U | NA | | Y | N | U | NA | | Y | N | U | NA | Y | | N | U | NA | |  |
| Araújo A. et al., 2016 |  | √ |  |  | √ |  |  |  | √ |  |  |  |  |  | √ |  | |  | √ |  |  | | √ |  |  |  | √ |  |  |  | | √ |  |  |  | | √ |  |  |  | |  |  |  |  |  | |  |  |  | | 7 |
| Dhavale H. et al., 2004 | √ |  |  |  |  | √ |  |  |  |  |  |  | √ |  |  |  | | √ |  |  |  | |  | √ |  |  | √ |  |  |  | | √ |  |  |  | | √ |  |  |  | |  |  |  |  |  | |  |  |  | | 6 |
| Duangrithi D.et al., 2016 | √ |  |  |  | √ |  |  |  | √ | √ |  |  |  | √ |  |  | |  | √ |  |  | | √ |  |  |  | √ |  |  |  | | √ |  |  |  | | √ |  |  |  | |  |  |  |  |  | |  |  |  | | 6 |
| Gebhardt S.et al., 2006 |  |  | √ |  |  | √ |  |  | √ |  |  |  | √ |  |  |  | |  | √ |  |  | | √ |  |  |  | √ |  |  |  | | √ |  |  |  | | √ |  |  |  | |  |  |  |  |  | |  |  |  | |  |
| Ghoreishizadeh M. and Deldoost F. 2008 | √ |  |  |  | √ |  |  |  |  |  |  |  | √ |  |  |  | | √ |  |  |  | |  | √ |  |  | √ |  |  |  | |  | √ |  |  | |  | √ |  |  | |  |  |  |  |  | |  |  |  | | 6 |
| Luft B. and Berent E., 2009 | √ |  |  |  | √ |  |  |  | √ |  |  |  | √ |  |  |  | | √ |  |  |  | | √ |  |  |  | √ |  |  |  | | √ |  |  |  | |  |  | √ |  | |  |  |  |  |  | |  |  |  | | 8 |
| Mentzel L., et al., 2017 | √ |  |  |  | √ |  |  |  | √ |  | √ |  | √ |  |  |  | | √ |  |  |  | | √ |  |  |  |  |  | √ |  | | √ |  |  |  | |  | √ |  |  | |  |  |  |  |  | |  |  |  | | 6 |
| Moreno-Calvete MC. 2013 | √ |  |  |  | √ |  |  |  | √ |  |  |  |  |  | √ |  | | √ |  |  |  | | √ |  |  |  | √ |  |  |  | | √ |  |  |  | | √ |  |  |  | |  |  |  |  |  | |  |  |  | | 7 |
| Ojagbemi A.et al., 2018 | √ |  |  |  | √ |  |  |  | √ |  |  |  |  |  | √ |  | |  | √ |  |  | | √ |  |  |  | √ |  |  |  | | √ |  |  |  | | √ |  |  |  | |  |  |  |  |  | |  |  |  | | 9 |
| Taye H.et al., 2014 | √ |  |  |  | √ |  |  |  | √ |  |  | √ | √ |  |  |  | |  |  | √ |  | | √ |  |  |  | √ |  |  |  | | √ |  |  |  | | √ |  |  |  | |  |  |  |  |  | |  |  |  | | 7 |
| Desai N. et al.2017 |  | √ |  |  | √ |  |  |  | √ |  |  |  | √ |  |  |  | | √ |  |  |  | | √ |  |  |  | √ |  |  |  | | √ |  |  |  | | √ |  |  |  | |  |  |  |  |  | |  |  |  | | 8 |
| Loughlin AM. et al., 2019 |  |  | √ |  | √ |  |  |  | √ |  | √ |  | √ |  |  |  | | √ |  |  |  | | √ |  |  |  | 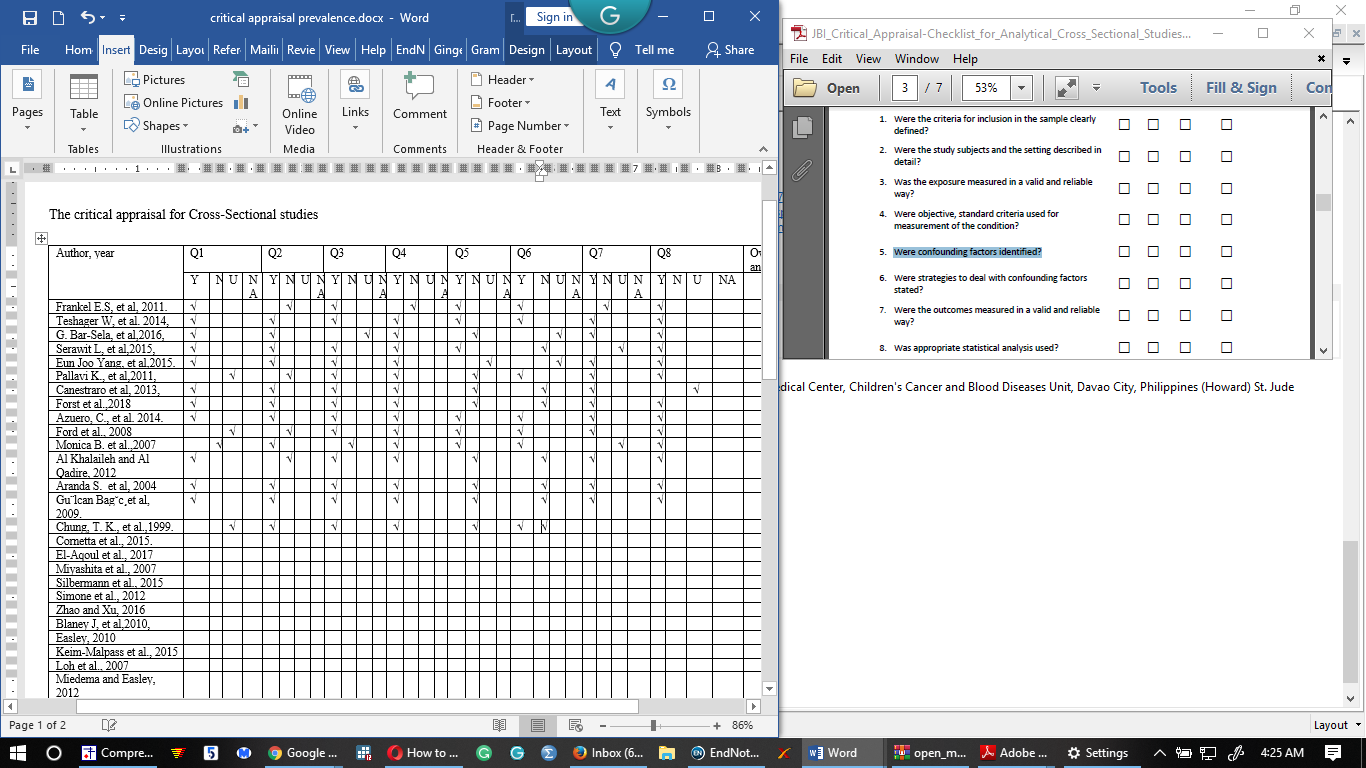 | √ |  |  | | √ |  |  |  | | √ |  |  |  | | √ |  |  |  | √ | |  |  |  | | 8 |
| Misdrahi D. et al., 2019 |  | √ |  |  | √ |  |  |  | √ |  |  |  |  |  | √ |  | |  | √ |  |  | | √ |  |  |  | √ |  |  |  | | √ |  |  |  | |  |  | √ |  | | √ |  |  |  | √ | |  |  |  | | 8 |
| Modestin J. et al., 2000 |  | √ |  |  | √ |  |  |  | √ |  |  |  |  |  | √ |  | |  |  | √ |  | | √ |  |  |  | √ |  |  |  | | √ |  |  |  | | √ |  |  |  | |  |  |  |  |  | |  |  |  | | 7 |
| Berardi D. et al., 2000 | √ |  |  |  | √ |  |  |  | √ |  |  |  | √ |  |  |  | |  |  | √ |  | | √ |  |  |  | √ |  |  |  | | √ |  |  |  | | √ |  |  |  | |  |  |  |  |  | |  |  |  | | 7 |

**JBI critical appraisal checklist for studies reporting prevalence data and cohort studies**

****Y=yes, N=no, U=unclear, NA=not applicable***
