# Supplementary material for: Antipsychotic-induced extrapyramidal side effects: A systematic review and meta-analysis of observational studies
Source: PLoS One. 2021 Sep 10;16(9):e0257129. doi: 10.1371/journal.pone.0257129 (PMC8432767; doi:10.1371/journal.pone.0257129)
Supplement: Attachment — Submitted filename: Response to reviewers.docx [file pone.0257129.s005.docx]

**Response to Academic Editor and reviewers**

**Dear,**

We would like to thank for your constructive comments to improve the manuscript titled “**Antipsychotic Induced Extrapyramidal Side Effects: A systematic review and meta-analysis**” (ID: **PONE-D-20-32247)**. Hereunder are our reaction to the academic editor and reviewers’ comments.

| **Comments and questions** | **Authors response** |
| --- | --- |
| **Academic Editor**  Please include the following items when submitting your revised manuscript:   - A rebuttal letter that responds to each point raised by the academic editor and reviewer(s). You should upload this letter as a separate file labeled ‘Response to Reviewers’. - A marked-up copy of your manuscript that highlights changes made to the original version. You should upload this as a separate file labeled ‘Revised Manuscript with Track Changes’. - An unmarked version of your revised paper without tracked changes. You should upload this as a separate file labeled ‘Manuscript’. | - An author response letter addressing comments forwarded from the academic editor and reviewer(s); revised manuscript with track changes that highlights changes made to the original version; and a clean version of the revised manuscript are separately presented as per the request. - Overall, the comments and suggestions provided by the academic editor is accepted and corrected accordingly. |
| **Reviewer 1** | |
| Reviewer #1: The authors have done a good job revising their manuscripts and improving the clarity of methods and results. I have no further comments | **Thank you, dear reviewer.** |
| **Reviewer 2** | |
| Reviewer #2: Comments 1. In the previous evaluations or comments the author(s) reacts for raised comments superficially. Or the author(s) was (were) not answer for reviewers’ comments. For example, EPSEs which is under introduction page 3 paragraph 1 line 5, and EPS which is on the same page last paragraph line 3 were not similar. Well if the author(s) incorporate deeply for the raised questions. | **Thank you for your constructive comments**   - The editorial error related to “EPS” and “EPSE” is corrected in the current version of the manuscript - As per your request, we have used abbreviations consistently throughout the document. We preferred to use EPSE - For terms which were repeated for at least three times, the full term with abbreviation in the parenthesis was used at the first appearance and the abbreviation only thereafter.   Kindly check the amendments (track-changes) made on this version |
| 2. Under results (search findings), the end line of first paragraph page 7, you were exclude 43 papers because various reason. Could explain these various reasons? | In the eligibility process, 43 articles were excluded for various reasons  such as studies that didn’t report the study outcome, having incomplete information, and/or those reported vague or mixed findings. |
| 3. The publication years of the included studies ranged from 2000 to 2019. I think it is too old the papers you were used from 2000. | - The following points are some of the reasons - We have considered methodological updates, time sensitivity and consistency of the research contents - To have a comprehensive search and extended coverage in terms of time and place. We considered the following points   - For instance, most of the measurement scales including Akathisia Rating Scale, Unified Parkinson Disease Rating Scale, Abnormal Involuntary Movement Scale (for TD) came in clinical practice in late 1980s and mid1990s.   - In the preliminary screening process, majority of studies about this research topic were conducted in the last 20 years (21^st^ century).   - Advances in pharmaceutical formulations in the area of antipsychotics should also be considered   - In our case, we finally included 60% studies from 2010-2019 and 40% from 2000-2009 after PRISMA flow chart and quality assessments.   - **Hence, it is not too old to include the articles published in the last two decades in this systematic review and meta-analysis. This would have been the major concern if the authors had considered the articles without time restriction.** |
| 4. Papers included in the study form both America and Africa is too few. This implies that as not enough studies were conducted with same title. Could you explain? | Dear reviewer, few numbers of articles included from America and Africa do not necessarily reflect the numbers of articles getting published from these continents. It is the PRISMA and quality assessments that determine their fate of inclusion and exclusion. There are several articles getting published in the area of antipsychotics and their side effects but the nature of the study (e.g., qualitative, in-vitro, preclinical, purely interventional, case report, case series, opinion, review etc…) maters. |
| 5. Title of the table needs modification. Table 1 page 9 | We have rephrased it as ‘Summary of studies included in the systematic review and meta-analysis’ |
| 6. An article you were used in the raw 10 column 2 is not clear. Which said South Africa and Nigeria | Dear reviewer,  This article is a multicenter study encompassing two African countries namely South Africa and Nigeria (patients presenting for biomedical treatment as out- or in-patients in psychiatric hospitals and community clinics in Cape-Town, South Africa and Ibadan Nigeria). We included it as it fulfills the eligibility criteria.  Here is the link of the study  [**https://www.ncbi.nlm.nih.gov/pmc/articles/PMC5946006/**](https://www.ncbi.nlm.nih.gov/pmc/articles/PMC5946006/) |
| 7. Please modify the strength and limitation of the study separately. It is not clear as it is. | Dear, we have put the strength and limitation sections sequentially as per the request |
| **Reviewer 3** | |
| Reviewer #3: Thank you for this informative review and would like you to consider the future focused review as mentioned before. | Thank you, dear reviewer. |
